# Efficacy of Eculizumab in a Patient With Immunoadsorption-Dependent Catastrophic Antiphospholipid Syndrome: A Case Report

**DOI:** 10.1097/MD.0000000000000143

**Published:** 2014-12-05

**Authors:** Andreas Kronbichler, Renate Frank, Michael Kirschfink, Ágnes Szilágyi, Dorottya Csuka, Zoltán Prohászka, Peter Schratzberger, Karl Lhotta, Gert Mayer

**Affiliations:** From the Department of Internal Medicine IV (Nephrology and Hypertension) (AK, PS, GM); Department of Radiology (RF), Medical University Innsbruck, Innsbruck, Austria; Institute of Immunology (MK), University of Heidelberg, Heidelberg, Germany; 3rd Department of Medicine (AS, DC, ZP), Research Laboratory, Faculty of Medicine, Semmelweis University, Budapest, Hungary; and Department of Nephrology and Dialysis (KL), Academic Teaching Hospital Feldkirch, Feldkirch, Austria

## Abstract

Catastrophic antiphospholipid syndrome (CAPS) is a rare but devastating complication in patients with antiphospholipid syndrome (APS) with a high morbidity and mortality.

We describe a case of a 30-year old female patient with immunoglobulin A (IgA) deficiency who underwent splenectomy because of idiopathic thrombocytopenic thrombocytopenia. Subsequently, an APS and finally systemic lupus erythematosus was diagnosed. After an uncomplicated pregnancy that was terminated by cesarean section, the patient developed severe CAPS with cerebral, myocardial, renal, and pulmonary involvement.

Because of IgA deficiency, standard therapy consisting of plasmapheresis and intravenous immunoglobulins in addition to steroids was not tolerated. After 8 sessions of immunoadsorption (IAS), massive pulmonary hemorrhage was controlled but relapsed twice whenever IAS was terminated. As other immunosuppressive agents were considered dangerous because of the risk of infections in the face of severe hypogammaglobulinemia, we administered eculizumab, an inhibitor of the terminal complement pathway, which led to a persistent control of her disease. Interestingly, eculizumab therapy was associated with a further decline of complement C3 and C4 serum levels. The patient developed a subsequent flare of her systemic lupus erythematosus, potentially indicating that complement inhibition by eculizumab is not effective in preventing lupus flares.

Taken together, we describe a unique case of life-threatening and difficult-to-treat CAPS with a good clinical response after terminal complement complex inhibition with eculizumab. Further controlled trials are necessary to investigate the value of eculizumab in patients with CAPS.

## INTRODUCTION

Catastrophic antiphospholipid syndrome (CAPS) is a potentially life-threatening and rare variant of the antiphospholipid syndrome (APS), characterized by vascular thrombosis in, among others, the brain, lung, heart, and kidney, ultimately leading to multiorgan failure. Most patients develop antiphospholipid antibodies and thrombocytopenia at the time of onset, whereas initially hemolytic anemia, disseminated intravascular coagulation, and the presence of schistocytes can be missing. Although diagnostic and therapeutic approaches improved over the last years, the morbidity and mortality of patients with CAPS is still high.^1^ Pregnancy and puerperium, per se predisposing to thrombotic events because of the induction of a procoagulatory state, are well-established triggers of the catastrophic variant,^2^ especially when complicated by preeclampsia. Mutations of complement regulatory proteins including membrane cofactor protein, complement factor I, and complement factor H have also been observed in patients with systemic lupus erythematosus (SLE) and antiphospholipid antibody positivity.^3^

## CASE REPORT

**FIGURE 1:**
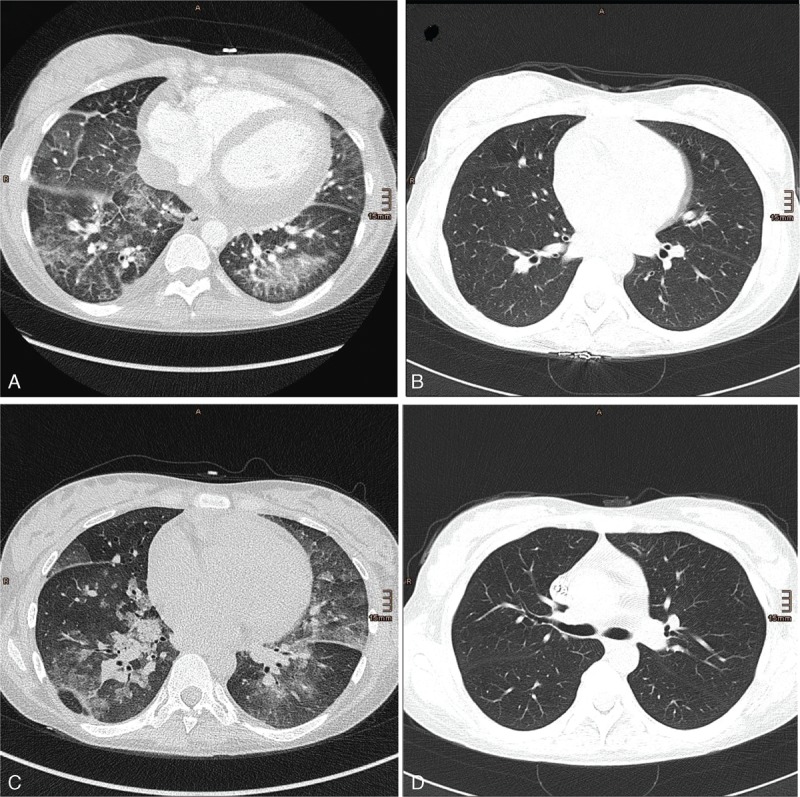
(A) Diffuse pulmonary hemorrhage in both lower lobes, which resolved after another initiation of (B) IAS. (C) After discontinuation of IAS, recurrence of pulmonary hemorrhage could be detected. These findings prompted us to initiate yet another series of IAS together with administration of eculizumab. (D) Complete resolution was detected in a control computed tomography 4 days later. IAS = immunoadsorption.

**FIGURE 2:**
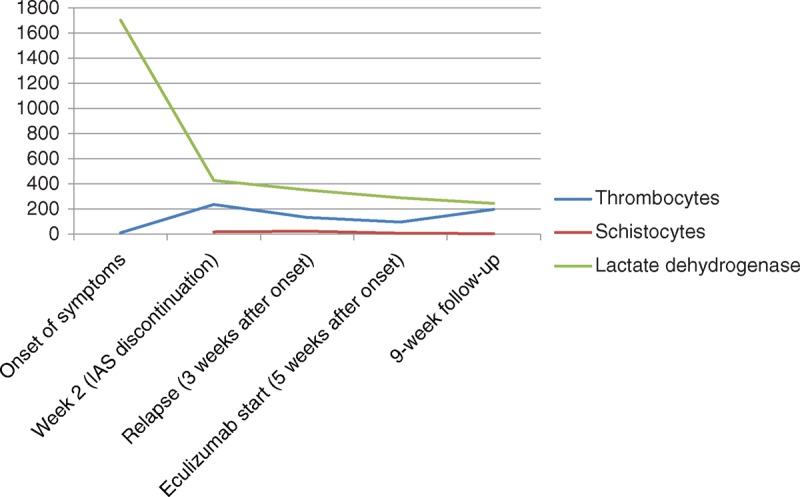
Laboratory values at the onset of the disease and at the time point of stable remission following eculizumab administration. Reference values of the respective parameters: thrombocytes (150–380 G/L), schistocytes (<5 per mille), and lactate dehydrogenase (100–250 U/L). Lactate dehydrogenase and schistocytes returned to normal values in the ninth week after onset time point and 4 weeks after initiation of eculizumab.

We report a 30-year-old woman, in whom splenectomy was necessary because of idiopathic thrombocytopenic thrombocytopenia in 1997. Primary APS was diagnosed in 2004 after onset of deep venous thrombosis with antibodies against anticardiolipin (>90 U/mL, immunoglobulin M [IgM] and immunoglobulin G [IgG] positive) along with anti-beta 2-glycoprotein (>90 U/mL), and she finally fulfilled the diagnostic criteria of SLE^4^ in 2010 with predominance of musculoskeletal and hematologic involvement. During her first pregnancy, she was on antimalarial therapy with chloroquine and low-molecular weight heparin because of APS. After cesarean section and delivery in April 2013, confusion, acute renal failure, myocardial ischemia with heart failure, severe thrombocytopenia, and hemolytic anemia attributed to CAPS developed. Dialysis was initiated and high-dose corticosteroid therapy including initial bolus methylprednisolone (250 mg daily for 3 days) followed by oral methylprednisolone (1.5 mg/kg body weight), rituximab (1 g with a repeated administration after 4 weeks), and plasmapheresis was started. Plasma exchange had to be stopped because of severe intolerance reactions, which were attributed to a selective immunoglobulin A (IgA) deficiency, which also precluded high-dose intravenous immunoglobulin therapy. The patient's condition deteriorated and she developed respiratory distress. A computed tomography scan showed diffuse alveolar hemorrhage (Figure 1A). Immunoadsorption (IAS) therapy using the Life 18 (Miltenyi Biotec, Bergisch Gladbach, Germany) was started with a total of 8 sessions. Treatment ameliorated thrombocytopenia and led to a resolution of the lung injury (Figure 1B). However, the patient was still dependent on dialysis. A renal biopsy revealed typical microangiopathic injury. After recurrence of pulmonary hemorrhage despite continuous high-dose methylprednisolone therapy, 10 additional daily IAS sessions were performed with clinical success. However, lung failure recurred again within 4 days after IAS withdrawal (Figure 1C) together with a rise in lactate dehydrogenase, thrombocytopenia, anemia, and a schistocyte count of 19 per mille. Thus, 4 additional sessions of IAS were necessary to control the disease again (Figure 1D). Due to low leukocyte counts and persistently low immunoglobulin levels (IgG 37 mg/dL and IgM 14 mg/dL, respectively), cytotoxic therapy was considered dangerous because of the risk for serious infections. It was, therefore, decided to administer eculizumab, a monoclonal antibody against the complement component C5, which prevents the activation of the terminal complement pathway. Within 4 days, respiratory failure completely resolved and signs of hemolytic anemia disappeared despite cessation of IAS. Finally, therapeutic anticoagulation with low molecular heparin could be commenced. The patient was discharged dialysis dependent, but with increasing amounts of urine 71 days after admission in July 2013 with a methylprednisolone dosage of 60 mg/day and on eculizumab treatment (weekly administration of 900 mg 4 times, followed by 1200 mg fortnightly). Laboratory values at the onset of the disease, after 3 weeks, at the time of eculizumab initiation, and after achievement of stable remission are depicted in Figure 2.

Two weeks after the discharge, the patient presented again with signs of hemolysis after the fourth and fifth eculizumab infusion. In addition, the serum levels of complement C3 and C4 were consistently reduced. Serum levels of both complement components further declined in the days following eculizumab infusion (data not shown). The patient received oral anticoagulation with acenocoumarol. Eculizumab concentrations measured prior and after application revealed efficacious serum concentrations, assured complete blockage of the terminal complement pathway, and neutralizing antibodies could not be detected. Addition of mycophenolate mofetil sufficiently abrogated hemolysis, which was finally attributed to activity of the underlying SLE, indicating a lack of efficacy of eculizumab in preventing a lupus flare in this patient. The application of eculizumab could be stopped after 3 months in September 2013 after a total of 9 infusions without recurrence of thrombotic microangiopathy despite a persisting positive test for antiphospholipid antibodies. Several measurements of C3d and the terminal complement complex (sC5bC9) while under treatment revealed normal serum levels (data not shown). Examination of the complement regulatory protein factors H and I as well as the alternative pathway component factor B revealed values within a normal range, indicative of a normal alternative complement pathway. However, persistently reduced C3 and C4 serum levels along with a low C1q (normal in one measurement and slightly reduced in another) are suspicious for a consumption of the classical complement pathway (Table 1). In addition, to exclude inherited predisposing factors in complement C3 or C4, genetic analysis was performed. A rare heterozygous synonymous variation in exon 13 of the *C3* gene (c.1677C>T; p.C559C) was detected, which was predicted as disease causing by MutationTaster (http://www.mutationtaster.org/) with a high probability (0.99) (Table 2). Analysis of C*4* revealed the presence of 2 copies of the C*4A* and 1 copy of the C*4B* genes (data not shown).

**TABLE 1:**
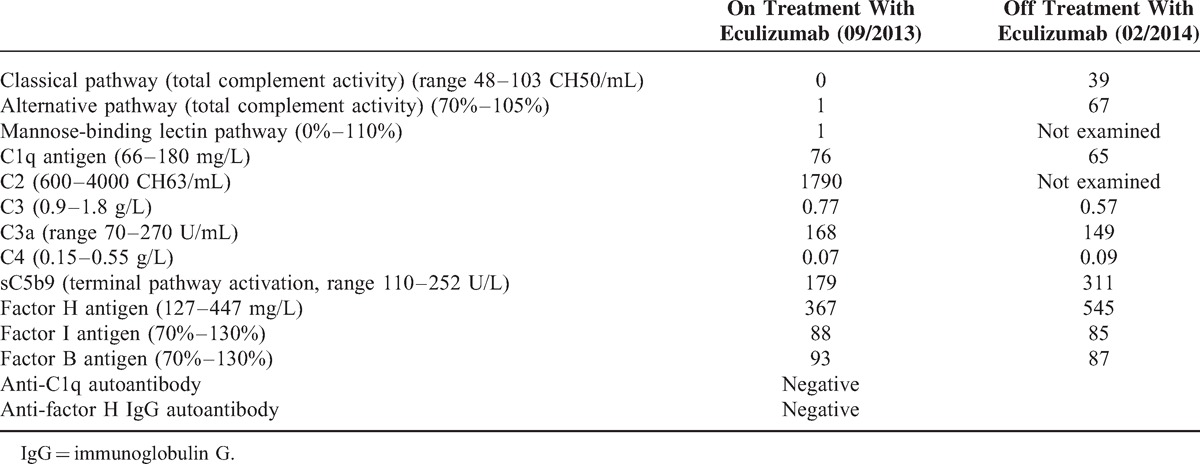
Complement Analysis After Application of Eculizumab and 6 mo After Cessation of Therapy

**TABLE 2:**
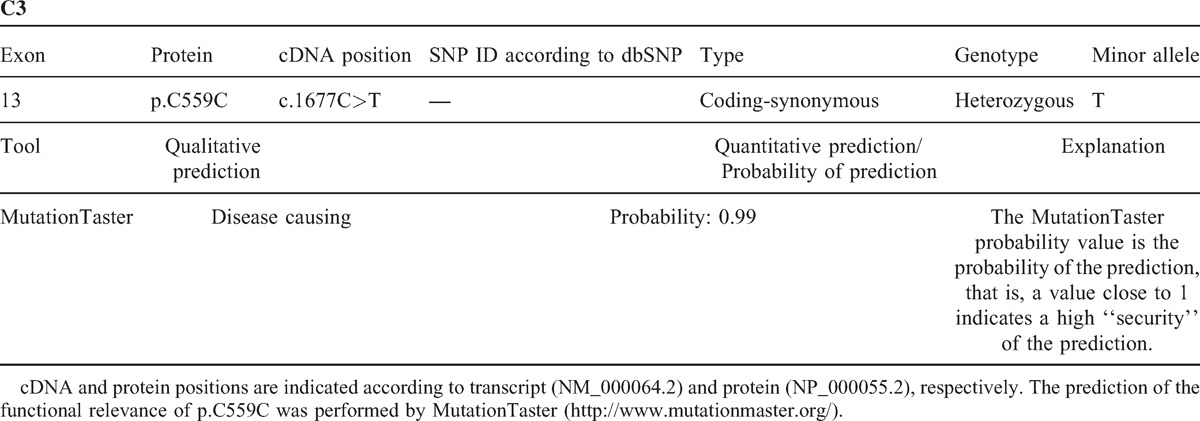
Genetic Analysis of Complement C3

One year after the initial insult, the patient is still dialysis dependent. Steroid reduction was well tolerated without signs of active thrombotic microangiopathy. The patient is listed for renal transplantation. Her current medication consists of mycophenolate mofetil 250 mg twice a day, methylprednisolone 10 mg every second day, blood pressure-lowering medication, diuretics, thyroid hormone substitution, and acenocoumarol with a target international normalized ratio of 2 to 3.

## DISCUSSION

Eculizumab has already shown encouraging results in a patient with recurrence of CAPS with reversal of thrombocytopenia and prevention of further clinical episodes of thrombosis.^5^ Its successful use has also been reported in recurrence of CAPS after renal transplantation.^6,7^ Transplantation in CAPS is also possible with prophylactic eculizumab administration.^8^ In addition, murine models reveal a pivotal role of the complement system in antiphospholipid antibodies-induced thrombosis along with endothelial cell and platelet activation. Prevention of terminal complement formation by using a monoclonal antibody against C5 inhibited thrombophilia induced by antiphospholipid antibodies.^9^ Complement activation and consumption was also confirmed in our patient by the finding that serum levels of C3 and C4 were significantly lowered while disease was highly active. Immunohistochemical analysis of the kidney biopsy specimen revealed a strong staining for C1q and IgM, while deposition of C3, IgG, and IgA was sparsely present. Interestingly, administration of eculizumab was associated with a further decrease in C3 and C4 serum levels. Moreover, sufficient eculizumab levels and complement inhibition was not capable of preventing a flare of her underlying SLE. Evidence that eculizumab may not be effective in SLE is still lacking, since there is limited data coming from a phase I single-center trial. No changes in laboratory values and systemic lupus erythematosus disease activity index have been observed in this preliminary clinical trial. However, the cohort of patients had low disease activity, thus precluding considerations on the therapeutic efficacy of eculizumab.^10^

Our case confirms previous reports that it is possible to discontinue eculizumab in CAPS after complete remission despite a continuing positive test for antiphospholipid antibodies. Persistently, reduced C4 and C3 levels in our patient can be explained by continuous activation of the classical complement pathway induced by immune complexes and antiphospholipid antibodies that cannot be influenced by eculizumab. Furthermore, a lowered C1q serum level after eculizumab cessation and strong deposition in the kidney biopsy also supports a consumption of the classical complement pathway. The finding that eculizumab application was followed by a further decrease in C4 and C3 serum levels is intriguing. One possible explanation could be that eculizumab interfered with clearance of immune complexes and thus caused a further stimulation of the classical pathway. Genetic analysis revealed a rare heterozygous variation in exon 13 of the C3 gene. This mutation does not cause an amino acid change in the C3 protein, so it is likely not causative; however, a potential influence cannot be excluded, as its predicted functional relevance showed a high probability as a disease-causing factor.

CAPS remains a severe variant of the APS with a high mortality rate of approximately 50%.^1^ However, patients achieving disease control are prone to a persisting morbidity as reported herein. This case is unique since the patient presented with a continuum of autoimmune disorders and had underlying IgA deficiency. Due to the latter, the standard therapy for CAPS was contraindicated or not tolerated by the patient. The patient had a good clinical response toward IAS combined with high doses of methylprednisolone, whereas she was refractory to rituximab. Inhibition of the terminal complement pathway by eculizumab led to a persistent clinical remission without evident recurrence of disease.

## CONCLUSION

Targeting complement activation may provide a new therapeutic option in the treatment of CAPS, especially in patients refractory or unable to tolerate standard therapy. In our exceptional case, stabilization of the disease course was possible after eculizumab was commenced. Further clinical investigations are necessary to clarify whether it can be generalized that blockade of the terminal complement activation is efficacious in the treatment of CAPS.
